# Dietary Administration of 2-Deoxy-D-Glucose Reduces High Fat Diet Induced Obesity and meta-inflammation in Mice

**DOI:** 10.1093/geroni/igaa057.3379

**Published:** 2020-12-16

**Authors:** Sanjay Pandey, Diksha Joshi, Saurabh Singh, Sushma Ray, Anant Narayan Bhatt, K Natarajan, B S Dwarakanath

**Affiliations:** 1 Institute of Nuclear Medicine and Allied Sciences, Delhi, Delhi, India; 2 Albert Einstein College of Medicine, Bronx, New York, United States; 3 Dr. B.R. Ambedkar Center for Biomedical Research, University of Delhi, Delhi, Delhi, India; 4 Shanghai Proton and Heavy Ion Center of Fudan University Shanghai Cancer Center, Delhi, Delhi, India

## Abstract

Obesity is a major risk factor for type 2 diabetes, NAFLD, chronic diseases and cancer. Insulin resistance, oxidative stress, high ectopic lipid levels and meta-inflammation are the mechanisms proposed to play a leading role in the morbidity associated with obesity. Energy restriction mimetics (ERMAs) has also been shown earlier to reduce the scale and the severity of these disorders by mimicking the physiological effects of the Energy Restriction. In present study we propose that the use of 2-DG as ERMA can be effective in regulating the High Fat Diet (HFD) induced obesity. Effect of 2-DG (0.4% w/v in drinking water) on the HFD and Insulin Resistance (IR). HFD induced change in body weight, adipose tissue mass, and ectopic lipid levels was assessed as the measure of obesity.IR and glucose levels were also estimated to evaluate the effect of 2-DG on the insulin sensitivity in HFD mice. 2-DG significantly altered HFD induced increase in the mice body weight, epididymal White Adipose Tissue (WAT) and liver weight. 2-DG fed mice also showed reduced lipid levels in serum and liver. Furthermore, 2-DG also reduced the oxidative damage in the liver with concomitant increase in enzymatic (SOD and Catalase) and non-enzymatic (reduced Glutathione) antioxidant levels. 2-DG fed mice also showed reduced levels of Leptin, IL-6 and TGF-β which are early drivers of the etiology of the metabolic diseases. Our results suggest that 2-DG as ERMA can prevent obesity and etiology of associated disorders. However, more relevant models are needed to further strengthen these observation

